# 

**Published:** 1882-07

**Authors:** 


### *TABLE OF CONTENTS FOR JULY, 1882.*

ORIGINAL COMMUNICATIONS. PAGE

ART. I.—The Advantages and the Dangers of Intrauterine Injections. By CHARLES W. PILGRIM, M.D. .....433
ART. II.—Dental Society Meetings. By W. C. BARRETT, M.D., D.D.S. .....436

CLINICAL REPORTS.

Case of Complete Intestinal Obstruction Caused by the Appendix Vermiformis in the Femoral Canal ; Operation and Recovery .....442

ORIGINAL TRANSLATIONS AND ABSTRACTS.

Syphilis and Locomotor Ataxia .....444
Pericementitis .....450
The Geographical and Climatic Relations of Pneumonia .....453
An Eye Protector for use with the Monocular Microscope .....454
The Proper Strength of Antiseptic Solutions to be Used in Surgery .....455
The Pathological Anatomy of Laryngo-tracheal Phthisis .....455

SELECTIONS.

On Simple Chancres of the Preputial Margin .....457
Gallstones Passed by an Infant .....464
The Pre Cancerous Stage of Cancer .....464
Hernia of the Ovary .....466
The Position of the Ovaries .....468
Tinea Versicolor in a Child .....469
Dupuytren's Contraction of the Fingers .....469
Multiple Cerebro-Spinal Sclerosis .....470
Uterine Hydatids in Virgins .....471
A Remarkable Wound of the Brain .....471
Waller and DeWatteville on the Electrotonus of Human Nerves .....472
Treatment of Uremia in Children by Pilocarpin .....473
Rheumatismal Pott's Disease .....473
The Nerve Element in Whooping Cough .....474
Delirium Tremens .....475
Bromide of Sodium and Epilepsy .....476
Arsenical Treatment of Chorea .....477
Albuminuria and Eclampsia During Pregnancy .....477
The Electric Brush in the Treatment of Locomotor Ataxia .....479
Papillary Growths of the Leg Preceding Cancer .....480
Partial Resection of the Urinary Bladder .....481
Paracentesis Cranii in Cases of Hydrocephalus .....482
Case of Vicarious Menstruation .....482
Significance of Albuminuria .....483
Relations of Tuberculosis and Scrofulosis .....484
Subcutaneous Section of Adhesions for Reduction of Old Dislocations of the Shoulder-joint .....485
Sulphurous Acid as a Disinfectant .....485

EDITORIAL DEPARTMENT.

The Publication of the Transactions of the American Medical Association .....486
The American Medical Association .....487
The Excessive "Heated Terms" .....488
"Dental Society Meetings" .....488
CURRENT NEWS AND OPINION .....489–491
REVIEWS AND BIBLIOGRAPHICAL NOTICES .....491–494

POPULAR SCIENCE DEPARTMENT.

Dentition in the Horse and Other Animals .....495
Fishy Curiosities .....499
The Discovery of Porcelain .....500
Earthquakes in 1881 .....501
A New Race of People in Russia .....502
The Census of Great Britain .....503

